# Neural signatures of heterogeneity in risk‐taking and strategic consistency

**DOI:** 10.1111/ejn.15476

**Published:** 2021-10-12

**Authors:** Josh Leota, Tobias Kleinert, Alex Tran, Kyle Nash

**Affiliations:** ^1^ Department of Psychology University of Alberta Edmonton Alberta Canada; ^2^ Institute for Mental Health Policy Research Centre for Addiction and Mental Health (CAMH) Toronto Ontario Canada

**Keywords:** EEG, frequency bands, neural traits, personality neuroscience, risk‐taking, strategic consistency

## Abstract

People display a high degree of heterogeneity in risk‐taking behaviour, but this heterogeneity remains poorly understood. Here, we use a neural trait approach to examine if task‐independent, brain‐based differences can help uncover the sources of heterogeneity in risky decision‐making. We extend prior research in two key ways. First, we disentangled risk‐taking and strategic consistency using novel measures afforded by the Balloon Analogue Risk Task. Second, we applied a personality neuroscience framework to explore why personality traits are typically only weakly related to risk‐taking behaviour. We regressed participants' (*N* = 104) source localized resting‐state electroencephalographic activity on risk‐taking and strategic consistency. Results revealed that higher levels of resting‐state delta‐band current density (reflecting reduced cortical activation) in the left dorsal anterior cingulate cortex and the left dorsolateral prefrontal cortex were associated with increased risk‐taking and decreased strategic consistency, respectively. These results suggest that heterogeneity in risk‐taking behaviour is associated with neural dispositions related to sensitivity to the risk of loss, whereas heterogeneity in strategic consistency is associated with neural dispositions related to strategic decision‐making. Finally, extraversion, neuroticism, openness, and self‐control were broadly associated with both of the identified neural traits, which in turn mediated indirect associations between personality traits and behavioural measures. These results provide an explanation for the weak direct relationships between personality traits and risk‐taking behaviour, supporting a personality neuroscience framework of traits and decision‐making.

## Graphical abstract

This study demonstrates that lower activation in the left dACC and the left DLPFC in the resting brain are associated with increased risk‐taking and decreased strategic consistency, respectively. Thus, heterogeneity in risk‐taking is associated with neural dispositions related to sensitivity to the risk of loss, and heterogeneity in strategic consistency is associated with neural dispositions related to strategic decision‐making. Both identified neural traits mediate indirect associations between personality traits and behaviour, supporting a personality neuroscience framework of traits and decision‐making.

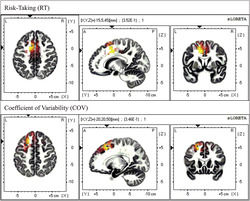

**Abbreviations**
AAHC: atomize agglomerate hierarchical clustering
ACC: anterior cingulate cortex
BA: Brodmann area
BART: balloon analogue risk task
CI: confidence interval
COV: coefficient of variability
DLPFC: dorsolateral prefrontal cortex
EEG: electroencephalography
fMRI: functional magnetic resonance imaging
MNI: Montreal Neurological Institute
PFC: prefrontal cortex
ROI: region of interest
RT: risk‐taking
SCS: self‐control scale
SE: standard error
sLORETA: standardized low resolution brain electromagnetic tomography
TIPI: Ten‐Item Personality Inventory
VMPFC: ventromedial prefrontal cortex

## 1 INTRODUCTION

People are highly heterogeneous in terms of risk‐taking. At the cultural level, heterogeneity in risk‐taking is relatively well characterized by macroeconomic variables, including ancient migration patterns, corporation and bank management, and cultural foundations (Becker et al., 2014, 2020; Laeven & Levine, 2009; Lee & Peterson, 2000; March & Shapira, 1987). At the individual level, heterogeneity in risk‐taking is not as well understood (l'Haridon et al., 2018; l'Haridon & Vieider, 2019). For example, individual differences in stable personality traits are only weakly and inconsistently associated with risk‐taking preferences (Becker et al., 2012), reflecting the general problem in psychological research that self‐reports are poorly predictive of behaviour (Dang et al., 2020). So, what leads one person to bold action and another to cautious restraint? Here, we used a neural trait approach to uncover the underlying neural sources of heterogeneity in risk‐taking. The neural trait approach examines links between task‐independent, brain‐based differences and behaviour in decision‐making or processes directly relevant to decision‐making (Baumgartner, Dahinden, et al., 2019; Brass & Haggard, 2007; Nash et al., 2015; Nash & Knoch, 2016; Schiller et al., 2014, 2020). Neural traits associated with certain functions can reveal the sources of behavioural heterogeneity and further suggest why people differ (for reviews, see Braver et al., 2010; Kanai & Rees, 2011; van den Heuvel & Hulshoff Pol, 2010). Frequency‐based measures of resting‐state EEG activity are heritable (Smit et al., 2005), specific to the individual (Dunki et al., 2000), and show high test–retest reliability (Dunki et al., 2000; Näpflin et al., 2007), thus acting much like a neural “fingerprint.”

Previous studies identified several major neural correlates of risk‐taking: The anterior cingulate cortex (ACC) is directly related to risky decision‐making in incentivized risk‐tasks (Eshel et al., 2007; Fishbein et al., 2005; Fukunaga et al., 2012; Paulus & Frank, 2006). For example, ACC activation increases during decisions against risk‐taking behaviour (Clark et al., 2008) and decreases during decisions to engage in further risk‐taking behaviour (Fukunaga et al., 2012), consistent with an inhibitory function in risky decision‐making. The ACC is crucially involved in error and performance monitoring (MacDonald et al., 2000), affecting sensitivity to the risk of loss by computing the likelihood and consequences of potential failure (Brown & Braver, 2007, 2008). Based on these studies, we assume that the ACC represents a primarily regulator of risk‐taking behaviour by inhibiting risky choices as part of a performance evaluation system. The insula is active during risky decision‐making as well, with a specific role in affective evaluations based on previous outcomes in sequential trials (risk prediction under uncertainty; Paulus et al., 2003; Preuschoff et al., 2008; for a review, see Singer et al., 2009). We therefore assume that the insula is related to both actual risk‐choices as well as strategic considerations based on previous experiences. Similarly, the amygdala is involved in affective processes related to gains and losses (e.g., Ernst et al., 2005), though less involved in the strategic decision‐making that we focus on in the current study (Ernst et al., 2002), which is why the amygdala is not further considered here. The dorsolateral prefrontal cortex (DLPFC) is related to risky decision‐making too. More specifically, upregulation or downregulation of the DLPFC leads to increased or decreased risk‐taking behaviour, respectively (Fecteau, Knoch, et al., 2007; Fecteau, Pascual‐Leone, et al., 2007; Figner et al., 2010; Knoch et al., 2006). However, previous research suggests that the DLPFC might be more indirectly involved in risky decision‐making by regulating long‐term or overall strategic considerations. For instance, it was found that strategic behaviour in economic decision games increased with age in children, that this increase could be explained by increased DLPFC activation, and that DLPFC thickness was positively correlated with strategic behaviour (Steinbeis et al., 2012). In another line of research, DLPFC activation increased during sequential risk‐decisions with increased uncertainty and thus higher strategic demands (Huettel et al., 2005; for a review, see Platt & Huettel, 2008). Furthermore, transcranial direct current stimulation of the DLPFC leads to better performance in a strategic Tower of London task (Dockery et al., 2009), and the DLPFC is more active during the strategic preparation of a subsequent Stroop task, whereas the ACC is more active during actual responding (MacDonald et al., 2000). All of this research is consistent with DLPFC roles in cognitive control and goal maintenance (MacDonald et al., 2000; Paxton et al., 2008; Silton et al., 2010). In line with previous findings, we assume that the DLPFC is primarily involved in regulating strategic considerations underlying risky decision‐making. Similarly, previous research suggests that the ventromedial PFC (VMPFC) is crucial for the basic encoding of value signals, which are later retrieved during actual risk‐taking behaviour (Hare et al., 2009). For example, increasingly risky choices in sequential trials were associated with decreased VMPFC activity (Schonberg et al., 2012), and VMPFC lesions can lead to nonstrategic gambling behaviour that does not take into account the odds of winning (Clark et al., 2008), consistent with a crucial role of the VMPFC for the strategic prevention of losses. The limited past research on neural traits of risk‐taking has typically correlated a single composite risk‐taking behavioural variable with source localized resting‐state EEG activation. In accordance with studies relying on event‐related analyses, risk‐taking relates to reduced activation in the dorsal PFC (Gianotti et al., 2012) and greater left‐than‐right lateral PFC activation at rest (Studer et al., 2013). Lateral PFC activation is inferred to reflect cognitive control or self‐regulation processes, whereas reduced activation in the dorsal PFC is inferred to reflect disinhibited and impulsive tendencies (Gianotti et al., 2009; Gläscher et al., 2012).

In the present study, we extended prior research in two key ways. First, we aimed to identify neural traits in EEG frequency bands of both risk‐taking and strategic consistency in the Balloon Analogue Risk Task (BART; Lejuez et al., 2002). Given that most people display a general tendency toward risk‐aversion (Kahneman & Tversky, 1979; Schonberg et al., 2012), higher risk‐taking scores might occur due to increased variability in responses rather than a general tendency for risky choices. By disentangling risk‐taking and strategic consistency, we aim to identify neural traits of both constructs, while previous neural trait research has not considered a separate role of strategy in risky decision‐making (e.g., Black et al., 2014; Gianotti et al., 2009, 2012; Gläscher et al., 2012; Santesso et al., 2008; Studer et al., 2013). Risk‐Taking (RT) is operationalized in the BART as the tendency to pump up balloons closer to an unknown explosion threshold, with every pump earning additional points, but also increasing the risk of losing points when causing an explosion. Notably, RT shows substantial heritability (Rao et al., 2018) and solid test–retest reliability (White et al., 2008), illustrating that it reflects a stable human trait. In addition to RT, we calculated a coefficient of variability (COV) as an inverse index of strategic consistency. COV is calculated as the standard deviation of pumps in the BART divided by average pumps (also see Bell et al., 2019; Blair et al., 2018; Congdon et al., 2013; DeMartini et al., 2014; Jentsch et al., 2010). A lower COV indicates consistent and strategic decision‐making, whereas a higher COV indicates less consistent and less strategic decision‐making. This assumption is based on the general notion that during sequential decision‐making in risk tasks, efficient strategies typically involve consistent decisions that are continuously adjusted in small steps based on previous outcomes (Pleskac, 2008; Wallsten et al., 2005; Zhou et al., 2021). In the BART, a consistent strategy would therefore be reflected by starting with an arbitrary number of pumps in the first trial, which would be slowly increased if balloons would not explode (e.g., 8 pumps ➔ 9 pumps ➔ 10 pumps ➔ 11 pumps …), resulting in a low COV and thus a high strategic consistency. On the other hand, an inconsistent strategy would be reflected by more random decisions (e.g., 5 pumps ➔ 15 pumps ➔ 2 pumps ➔ 18 pumps …), resulting in a high COV and thus less strategic consistency. In line with these considerations, a higher COV in the BART has been associated with lower age and lower executive function capacity in school‐age children (Bell et al., 2019), lower working memory capacity (Blair et al., 2018), lower functional (but not dysfunctional) impulsivity (Congdon et al., 2013), and a suboptimal strategy to gain rewards (Blair et al., 2018; DeMartini et al., 2014; Jentsch et al., 2010).

Second, we applied a personality neuroscience framework (DeYoung & Gray, 2009) to explore why personality traits are typically only weakly related to risk‐taking preferences (Becker et al., 2012). According to this framework, personality traits are assumed to show indirect associations with certain decisions and behaviours to the degree that these traits are associated with relevant neural systems. Essentially, this is a personality ➔ brain function ➔ behaviour perspective (also see Declerck et al., 2013). Here, we tested if neural traits would mediate indirect associations of the Big 5 and trait self‐control with risk‐taking and strategic consistency. We chose the Big 5 because it is the most consistently used personality framework in risk‐taking research (Nicholson et al., 2005; Zuckerman & Kuhlman, 2000) and trait self‐control due to its strong relationship with risky behaviours (De Ridder et al., 2012; Tangney et al., 2004).

Based on previous research, we hypothesize that RT will be related to lower activation of the ACC (indicated by a higher current density in alpha/theta/delta frequency bands and/or a lower current density in beta frequency bands), whereas COV will be related to lower activation of the DLPFC and the VMPFC (indicated by a higher current density in alpha/theta/delta frequency bands and/or a lower current density in beta frequency bands). We further expect that both RT and COV will be related to stronger activation in the insula. Finally, we expect that indirect associations of the Big 5 and trait self‐control with RT and COV will be mediated by the respective neural traits identified in the primary analyses.

## 2 METHOD

### 2.1 Participants

Ethics approval for this study was provided by the University of Alberta Human Research Ethics Board; 110 participants were recruited for this study, from which six were excluded due to insufficient quality of resting EEG recordings, leaving a total sample of *N* = 104 for all analyses (mean age = 19.78; age range = 17–26; females = 61). Females and males showed nonsignificant differences concerning the main findings of this study, and consequently these results were not considered further (though see Table S3 for analyses involving gender). Participants were first year psychology students, had normal or corrected‐to‐normal vision, and were compensated for their time with class credit. We aimed to include 100 participants and stopped collection at the end of the 2019 fall term. An a priori power analysis for correlations in G*Power (Faul et al., 2009) yielded a required sample size of 64 participants (*α* = 0.05, *power* = 0.80, expected effect size of *r* = 0.30, based on Gianotti et al., 2012; Studer et al., 2013).

### 2.2 Procedure

After providing written informed consent, participants were equipped with a 64‐channel EEG system (Brain Products GmbH, Munich, Germany) and seated in an electrically and noise‐shielded cabin. All tasks were completed on a computer using the software Presentation (Version 18.0, Neurobehavioral Systems, Inc., Berkeley, CA). The data used to generate our findings are freely available in the Mendeley data repository (Kleinert, 2021; https://doi.org/10.17632/96bwxvb83m.1). All code and additional materials of this study are available upon request. First, participants provided demographic information and answered several questionnaires on personality traits, including the Big 5 personality traits and trait self‐control. Next, resting EEG was recorded for 4 min using a protocol that consists of a 1‐min eyes‐open period followed by a 1‐min eyes‐closed period, repeated two times in total. Alternating eyes‐open and eyes‐closed periods are routinely used in resting EEG research in order to achieve more stable mental states (Barry et al., 2007; Baumgartner, Langenbach, et al., 2019; Schiller et al., 2014, 2019, 2020), as participants can become drowsy during eyes‐closed periods after only a few minutes (Tagliazucchi & Laufs, 2014). In line with standard procedures for investigations of resting‐state brain activity (e.g., Damoiseaux et al., 2006; Mantini et al., 2007; for reviews, see Lee et al., 2013; Newson & Thiagarajan, 2019), only eyes‐closed periods were used for further analysis (2 min in total). The usage of eyes‐closed periods provides a more stable and more consistent measure of resting‐state brain activity compared with eyes‐open periods, as spontaneous processing of surrounding visual (and possibly emotional) stimuli do not affect the EEG (Barry et al., 2007).

As part of our broader research paradigm, participants were then randomly assigned to one of two experimental conditions (anxiety or control). Critically, controlling for this manipulation had no impact on the current results (all effects remain significant, and at similar levels; see Table S4). Participants then completed two tasks that are unrelated to the current study (a passive auditory oddball task and a Stroop task), followed by the BART, our behavioural measure of risk‐taking and strategic consistency (average duration of 3 min). Note that this study constitutes a reanalysis of BART behaviour (Nash et al., 2020) but focuses on the links with resting state EEG that are not reported elsewhere. Finally, participants were debriefed and compensated with class credit for their participation. On average, the duration of the whole experimental session was 110 min.

### 2.3 Balloon Analogue Risk Task

Risk behaviour as measured with the BART is associated with sensation seeking, impulsivity, and self‐control deficiencies. Furthermore, it is associated with addictive, health, and safety risk behaviour (Lejuez et al., 2002, 2003). Participants were told they would be entered into a lottery to win $100 and that they could increase their number of ballots (and chance of winning the money) by doing well on an online balloon‐pumping game. They then proceeded to the main task in which they pressed the space bar to inflate 20 balloons one after another, which had variable and unknown explosion thresholds. The average explosion threshold across trials was 15 pumps. Each pump slightly inflated the balloon and earned one point but also brought the balloon closer to the explosion threshold. Exploded balloons earned no points and participants could stop pumping at any time for each of the 20 balloon trials, retain the points earned for that balloon, and move on to the next trial. We computed a score of RT for each individual that increases with a higher average number of pumps and a higher number of explosions. RT was calculated as average pumps * (explosions + 1)/total number of trials (i.e., 20 trials). This score takes into account the influence of explosions (rather than using only trials without explosions) in order to achieve a more valid measure of risk‐taking, given that explosions can arguably result from risky behaviour in the BART. Note that results are highly similar using different computations of risk‐taking (e.g., total pumps or average pumps).

We computed a second measure from the BART to tease apart risk‐taking and strategic consistency. The COV is computed as the standard deviation of pumps across trials divided by average pumps across trials and therefore represents an inverse index of strategic consistency. A lower COV reflects a more consistent strategy in the BART. Conversely, a higher COV reflects a more inconsistent strategy in the BART. Given that the calculation of both RT and COV are based on the same decisions in the BART, we expect that they share some common variance. However, high levels of RT should result from consistently high‐risk choices, whereas high levels of COV should result from alternating high‐ and low‐risk choices, which is why both constructs should also cover unique variance.

### 2.4 Personality measures

As part of a larger package of personality questionnaires, participants completed the Ten‐Item Personality Inventory (TIPI; Gosling et al., 2003), which measures the Big 5 personality traits Extraversion, Agreeableness, Conscientiousness, Neuroticism, and Openness to Experience with two items per subscale. We chose the TIPI over other measures of the Big 5 in order to limit time spent on questionnaires to avoid nonconscientious responding due to inattention (Gosling et al., 2003). Items were rated on 5‐point Likert scales ranging from 1 (*strongly disagree*) to 5 (*strongly agree*). Although internal consistencies are low in the TIPI due to the small number of items (mean Cronbach's *α* = 0.55), subscales display substantial test–retest reliability after 6 weeks (mean *r* = 0.72), high convergence with an established measure of the Big 5 (mean *r* = 0.76) and expected patterns of external correlations (Gosling et al., 2003).

Participants also completed a short form of the Self‐Control Scale (SCS; Tangney et al., 2004), which measures Trait Self‐Control using 13 items. Items were rated on 5‐point Likert scales ranging from (1 = *strongly disagree*) to 5 (*strongly agree*). The SCS displays good internal consistency (Cronbach's *α* = 0.83 and 0.85 in two studies) and good test–retest reliability after 3 weeks (*r* = 0.87; Tangney et al., 2004). Furthermore, it is associated with higher grade point average, less psychopathology, higher self‐esteem, better relationships and interpersonal skills, secure attachment, and more functional emotional responses (Tangney et al., 2004).

### 2.5 EEG recording and preprocessing

Continuous resting EEG was recorded with a 64‐channel ActiCHamp system using Ag/AgCl electrodes (Brain Products GmbH, Munich, Germany) that were positioned according to the 10/10 montage. The EEG was online band‐pass filtered between 0.1 and 100 Hz and digitized with a sampling rate of 512 Hz (24‐bit precision). Electrode TP9, positioned over the left mastoid, served as the reference electrode during recording. Preprocessing was conducted using the BrainVision Analyzer software (Version 2.0.3 Brain Products GmbH, Munich, Germany). First, the EEG was re‐referenced to an average of the electrodes TP9 and TP10 (left and right mastoid electrodes) and down‐sampled to 256 Hz. Next, filters were applied (0.1‐ to 30‐Hz band pass filter, 60‐Hz notch filter). Resting‐state EEG recorded during the two 1‐min eyes closed measurements were then extracted for analysis. Eye movement artefacts were removed using the automatic ocular correction method by Gratton et al. (1983). Remaining artefacts were detected and removed automatically (criteria: −100 to +100 μV min/max threshold, 50 μV maximum voltage step, 0.5 μV lowest allowed voltage [maximum–minimum] in intervals of 100 ms).

### 2.6 EEG frequency bands and source localization

For frequency band analysis, epochs of 2 s were segmented from artefact‐free resting EEG (eyes‐closed segments only). Fast Fourier transform was used to calculate power spectra in each epoch using a hamming window, and epochs were allowed to overlap by 75% (allowing for substantial correlations among adjacent windows, but maximizing equal weighting of EEG epochs and thus minimize the loss of data) (Harris, 1978; for a review, see Pardey et al., 1996). For each individual, all available artefact‐free EEG epochs were used to compute average cross‐spectral matrices, and then average power values (μV^2^) for seven brain frequencies (delta: 1.5–6 Hz, theta: 6.5–8 Hz, alpha1: 8.5–10 Hz, alpha2: 10.5–12 Hz, beta1: 12.5–18 Hz, beta2: 18.5–21 Hz, beta3: 21.5–30 Hz; Henry, 2005; Kubicki et al., 1979). In line with previous research, we assume that slow‐wave frequency bands, such as delta, theta, and alpha1, reflect lower cortical activation, whereas fast‐wave frequency bands, such as beta, reflect higher cortical activation (for reviews, see Amzica & Steriade, 1998; Jensen & Mazaheri, 2010; Kilner et al., 2005; Klimesch, 1999; Laufs, 2008). Largely based on studies that linked fMRI BOLD activity to EEG frequency bands (e.g., Goldman et al., 2002; Laufs, Kleinschmidt, et al., 2003; Laufs, Krakow, et al., 2003), this heuristic model suggests that increased energy dissipation from neuronal membranes and increased coupling of neural assemblies are associated with both increased haemodynamic brain activation as well as a shift in spectral oscillations from slower to faster oscillations (for a review, see Kilner et al., 2005).

In order to estimate the cortical sources of absolute power in each of the resting EEG's seven frequency bands, we used standardized low‐resolution brain electromagnetic tomography (sLORETA; Pascual‐Marqui, 2002). This method computes activity as current density (A/m^2^) without assuming a predefined number of active sources. The solution space of sLORETA consists of 6239 voxels (voxel size: 5 mm^3^) covering cortical grey matter and hippocampi, as defined by the digitized Montreal Neurological Institute (MNI) probability atlas. For each individual, the computed sLORETA images were normalized (total current density = 1) and log‐transformed prior to analyses. sLORETA has been established as a standard method for EEG source localization (for a review, see Grech et al., 2008), demonstrating high long‐term reliability (Cannon et al., 2012; Tenke et al., 2017). Neural sources identified with sLORETA in resting‐state EEG data have been reliably associated with interindividual differences in risk‐preferences (Gianotti et al., 2009; Jäncke et al., 2008; Studer et al., 2013), social preferences (Baumgartner et al., 2013; Gianotti et al., 2019; Knoch et al., 2010; Schiller et al., 2019; for a review, see Nash et al., 2015) and imprudent choice (Gianotti et al., 2012), ideally qualifying the technique for neural trait research. Furthermore, brain activity identified by sLORETA is highly consistent with activity identified using other techniques, including functional magnetic resonance imaging (fMRI; Mobascher et al., 2009; Olbrich et al., 2009), Positron emission tomography (PET; Laxton et al., 2010) and intracranial recordings using implanted electrodes (Zumsteg et al., 2006). To further demonstrate the reliability of sLORETA in our own data, we split the resting‐state EEG in four segments of 30 s each and extracted estimates in a test ROI in the anterior cingulate cortex (BA 24 and 32, average across all voxels). Reliability analyses showed excellent internal consistency of current density across all frequency bands (all Cronbach's *α* > 0.9).

### 2.7 Statistical analyses

To determine the underlying neural sources of heterogeneity in risk‐taking and strategic consistency, whole brain voxel‐by‐voxel regression analyses were conducted separately for each EEG frequency, with resting‐state eyes‐closed sLORETA images as the dependent variable and the BART measures RT and COV as the independent variables. Correction for multiple testing for all 6239 voxels was implemented by means of a nonparametric randomization approach (Nichols & Holmes, 2002). This approach estimates empirical probability distributions and the corresponding critical probability thresholds (corrected for multiple comparisons).

Additionally, we applied an exploratory personality neuroscience framework (DeYoung & Gray, 2009) to investigate sources of heterogeneity in risk‐taking and strategic consistency in personality traits, including the Big 5 and trait self‐control. Specifically, we examined associations of personality traits with RT and COV. Next, we examined associations of personality traits with the neural traits linked to RT and COV that were identified in the preceding analyses. Finally, we used mediation models in the Process macro for SPSS (model 4, 10,000 bootstrap samples; Hayes, 2012) to test if neural traits mediate indirect associations between personality traits and the behavioural measures RT and COV (personality traits ➔ neural traits ➔ risk‐taking and strategic consistency).

## 3 RESULTS

### 3.1 Neural signatures of risk‐taking and strategic consistency

Participants showed considerable heterogeneity in risk‐taking (RT: *M* = 3.91, *SD* = 2.72, range = 0.13 to 12.68) and strategic consistency (COV: *M* = 0.47, *SD* = 0.13, range = 0.16 to 0.89) in the BART (for descriptive statistics of all main variables of the study, see Table S1). RT and COV were only marginally associated with one another (*r* = 0.165, *p* = 0.094), which fits well with previous research showing inconsistent associations of the two constructs (Blair et al., 2018; Congdon et al., 2013; DeMartini et al., 2014). However, they did show some shared variance, which led us to conduct commonality analyses (Nimon et al., 2008), subsequent to our main analyses, to disentangle independent associations of the two variables with the neural traits identified in this study.

**FIGURE 1:**
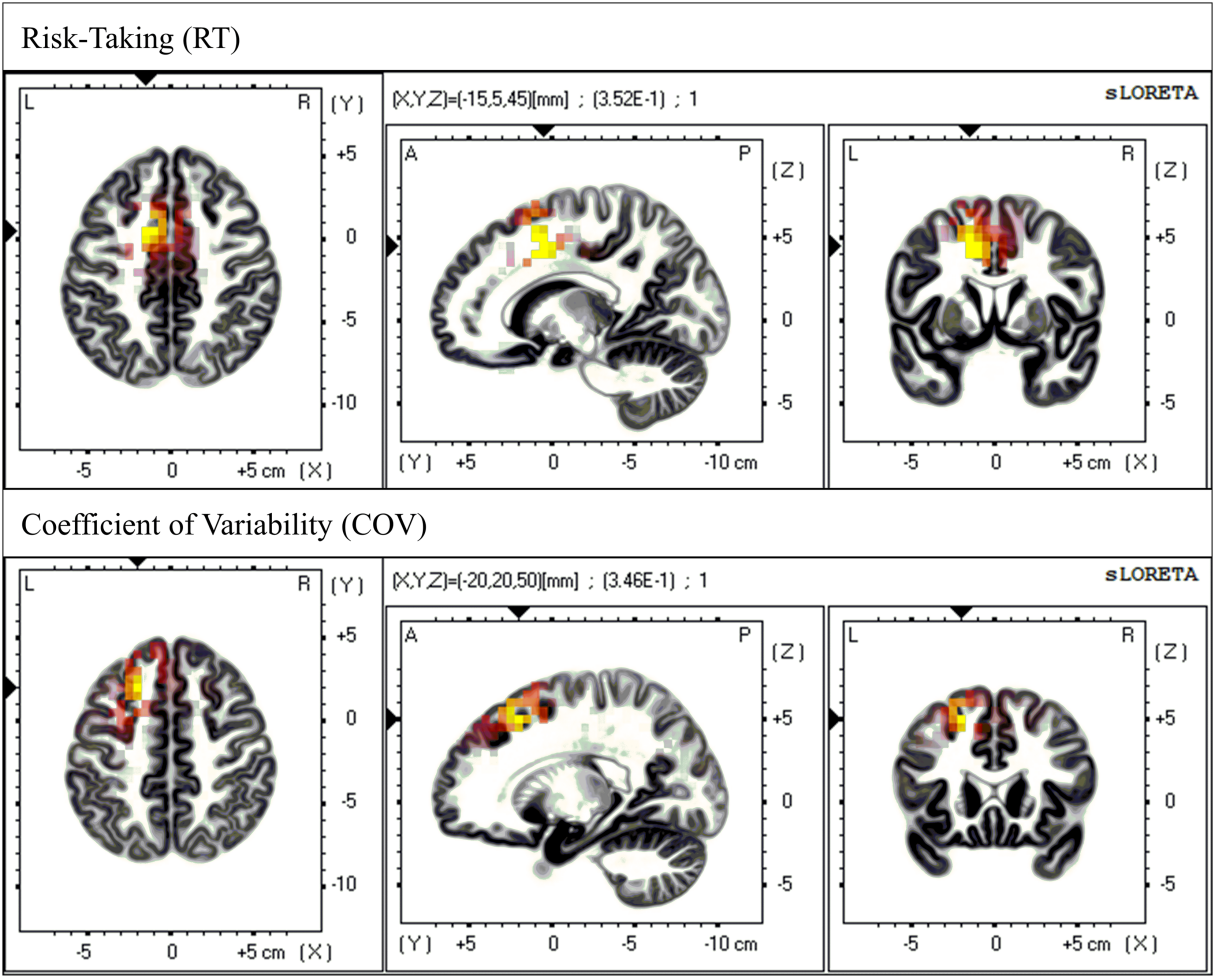
Neural sources of risk‐taking and strategic consistency in the delta frequency band at rest. Results from whole‐brain corrected, voxel‐by‐voxel regression analyses in sLORETA (Pascual‐Marqui, 2002). Coloured voxels indicate significant correlations of resting‐state current density in the delta frequency band with Risk‐Taking (RT) and strategic consistency as measured by the Coefficient of Variability (COV). RT was associated with current density in the delta band in the left dorsal ACC (MNI coordinate peak voxel: *x* = −15, *y* = 5, *z* = 45, Brodmann area 32; *r* = 0.352, corrected *p* < 0.05; all six voxels in the cluster: *r* = 0.344, corrected *p* < 0.05). COV was associated with current density in the delta band in the left DLPFC (MNI coordinate peak voxel: *x* = −20, *y* = 20, *z* = 50, Brodmann area 8, *r* = 0.346, corrected *p* < 0.05; seven contiguous voxels in the cluster: *r* = 0.324, corrected *p* < 0.10)

To test our main hypotheses, we conducted whole‐brain corrected, voxel‐by‐voxel regression analyses to determine the relationship between the separate behavioural indices of RT and COV and resting‐state brain activity in each frequency band (see Figure 1). Results showed that RT was associated with current density in the delta band in a cluster of six contiguous voxels in the left dorsal ACC (MNI coordinate peak voxel: *x* = −15, *y* = 5, *z* = 45, Brodmann area 32; *r* = 0.352, corrected *p* < 0.05; all voxels in the cluster: *r* = 0.344, corrected *p* < 0.05). On the other hand, COV was associated with current density in the delta band in a cluster of seven contiguous voxels in the left DLPFC (MNI coordinate peak voxel: *x* = −20, *y* = 20, *z* = 50, Brodmann area 8, *r* = 0.346, corrected *p* < 0.05; all voxels in the cluster: *r* = 0.324, corrected *p* < 0.10). As resting slow‐wave delta oscillations reflect reduced cortical activation (for reviews, see Amzica & Steriade, 1998; Kilner et al., 2005; Laufs, 2008; Riedner et al., 2011), these results suggest that increased risk‐taking is reflected by a decreased cortical activation in the left dorsal ACC, and decreased strategic consistency (i.e., higher COV) is reflected by a decreased activation in the left DLPFC. To disentangle independent associations of RT and COV with neural traits, we conducted commonality analyses (Nimon et al., 2008), using RT and COV as joint predictors for brain activity in the left ACC and the left dorsal DLPFC. Results show that RT uniquely accounted for 58.2% of the total variance explained in left dorsal ACC activation (vs. 26.18% for COV; shared variance = 15.62%), whereas COV uniquely accounted for 59.01% of the total variance explained in left DLPFC activation (vs. 25.46% for RT; shared variance = 15.53%). In sum, heterogeneity in risk‐taking and strategic consistency can thus be explained by neural traits in the left dorsal ACC and the left DLPFC, respectively (see sLORETA analyses controlling for multiple testing). Although both RT and COV were associated with both neural traits in simple correlation analyses that did not correct for multiple testing (see Table S2), subsequent commonality analyses illustrate that the majority of variance explained in left dorsal ACC activation is accounted for by RT, and the majority of variance explained in left DLPFC activation is accounted for by COV.

### 3.2 Neural signatures link personality traits to risk‐taking and strategic consistency

Finally, we applied a personality neuroscience framework (DeYoung & Gray, 2009) to examine the idea that personality traits may be more tightly linked with behavioural measures by incorporating the identified neural traits that explain heterogeneity in risk‐taking and strategic consistency. First, we correlated RT and COV with Big 5 and Trait Self‐Control personality traits, which were related to decision‐making in past research (albeit tenuously and inconsistently, see Becker et al., 2012). We found similarly weak relationships. Only Extraversion was correlated with RT (*r* = 0.234, *p* = 0.017) and COV (*r* = 0.224, *p* = 0.022).

We next extracted individual estimates of current density in the delta band across voxels in a 10‐mm sphere in the left dorsal ACC around the peak voxel that correlates with RT (MNI coordinate peak voxel: *x* = −15, *y* = 5, *z* = 45) and in the left DLPFC around the peak voxel that correlates with COV (MNI coordinate peak voxel: *x* = −20, *y* = 20, *z* = 50). Delta band activation in the left dorsal ACC was broadly correlated with Big 5 personality traits, including Extraversion (*r* = 0.313, *p* = 0.001), Conscientiousness (*r* = 0.229, *p* = 0.020), Neuroticism (*r* = −0.276, *p* = 0.005), Openness to Experience (*r* = 0.281, *p* = 0.004), and Trait Self‐Control (*r* = 0.224, *p* = 0.022). Additionally, delta band activation in the left DLPFC was correlated with personality trait measures, including Extraversion (*r* = 0.222, *p* = 0.024) and Openness (*r* = 0.228, *p* = 0.020). For all correlations of personality traits and behavioural measures with neural traits, see Table S2.

**FIGURE 2:**
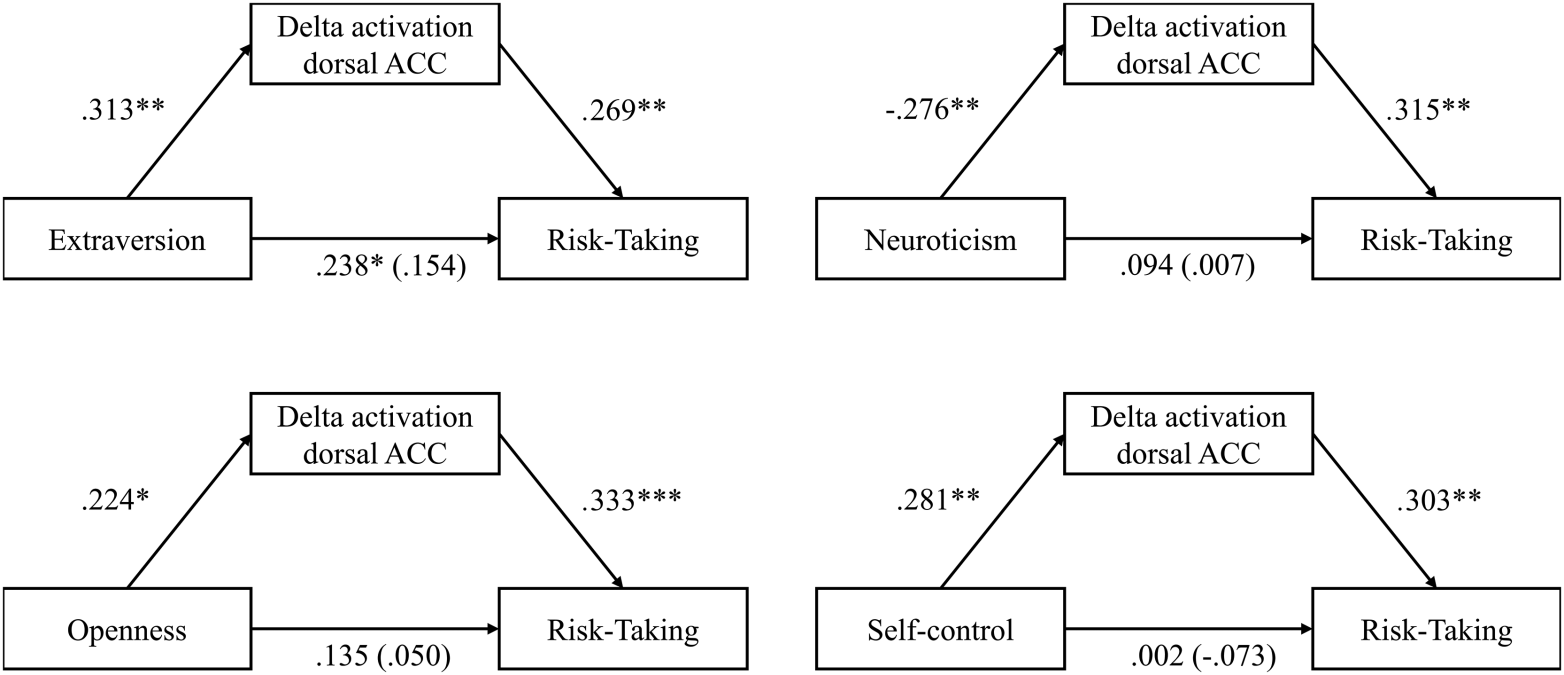
Delta activation of the left dorsal ACC mediates indirect effects of personality traits on risk‐taking. ^*^ *p* < 0.05, ^**^ *p* < 0.01, ^***^ *p* < 0.001. Mediation models illustrating that delta activation in the left dorsal ACC (mediator = M) mediates indirect effects of Extraversion, Neuroticism, Openness to Experiences and Self‐Control (iV = X) on risk‐taking (dV = Y), supporting a personality neuroscience framework of traits and decision‐making. Shown are standardized regression coefficients obtained from Process (Hayes, 2012) for the associations between X and M and for the associations between M and Y. Total effects and direct effects controlling for M (in parentheses) are shown for associations between X and Y. In all models, significant proportions of the effects between X and are mediated through M

**FIGURE 3:**
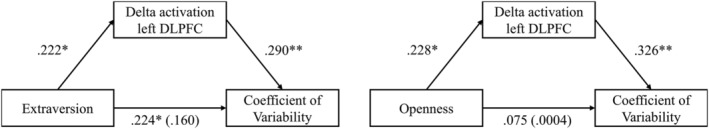
Delta activation of the left DLPFC mediates indirect effects of personality traits on strategic consistency. ^*^ *p* < 0.05, ^**^ *p* < 0.01, ^***^ *p* < 0.001. Mediation models illustrating that delta activation in the left DLPFC (mediator = M) mediates indirect effects of Extraversion and Openness to Experiences (iV = X) on strategic consistency as measured with the Coefficient of Variability (COV; dV = Y), supporting a personality neuroscience framework of traits and decision‐making. Shown are standardized regression coefficients obtained from Process (Hayes, 2012) for the associations between X and M and for the associations between M and Y. Total effects and direct effects controlling for M (in parentheses) are shown for associations between X and Y. In both models, significant proportions of the effects between X and are mediated through M

Lastly, mediation analyses in Process (model 4, unstandardized coefficients, 10,000 bootstrap samples; Hayes, 2012) revealed that left dorsal ACC activation mediated an indirect effect of personality trait on RT for Extraversion (*indirect effect coefficient* = 0.217, SE = 0.109, 95% CI = 0.040, 0.465), Neuroticism (*indirect effect coefficient* = −0.247, SE = 0.109, 95% CI = −0.481, −0.055), Openness to Experience (*indirect effect coefficient* = 0.304, SE = 0.131, 95% CI = 0.083, 0.595), and Trait Self‐Control (*indirect effect coefficient* = 0.308, SE = 0.175, 95% CI = 0.012, 0.687; see Figure 2 for mediation models). Analogous analyses revealed that left DLPFC activation mediated an indirect effect of personality traits on COV for both Extraversion (*indirect effect coefficient* = 0.008, SE = 0.004, 95% CI = 0.018, 0.001) and Openness to Experience (*indirect effect coefficient* = 0.013, SE = 0.006, 95% CI = 0.027, 0.003; see Figure 3 for mediation models). Together, these results support a personality neuroscience framework (DeYoung & Gray, 2009) in which broad personality traits predict behaviour indirectly to the extent that they predict neural traits associated with heterogeneity in risk‐taking and strategic consistency.

## 4 DISCUSSION

People display a significant degree of heterogeneity in risk‐taking, but the sources of such heterogeneity are yet poorly understood. Here, we used a neural trait approach to help characterize people who differ in risk‐taking and strategic consistency and reveal the neural sources of those behavioural differences. Further, we extended prior research on neural traits of risk‐taking behaviour (Gianotti et al., 2009) in two key ways. First, we measured both risk‐taking and strategic consistency in the BART. Second, we applied a personality neuroscience framework (DeYoung & Gray, 2009) to examine if personality traits may relate to risk‐taking and strategic consistency to the degree that they are related to the underlying neural traits.

As hypothesized, we found that higher levels of risk‐taking were associated with decreased resting‐state activation in the left dorsal ACC. We found no significant associations with insular activation. The dorsal ACC is broadly associated with performance monitoring, sensitivity to negative outcomes, and conflict detection processes (Brown & Braver, 2007, 2008; Carter et al., 1998; Holroyd et al., 2004; MacDonald et al., 2000; Van Veen et al., 2001). Importantly, a previous study shows that dorsal ACC activation tracks increasing risk‐taking across trials in the BART (Rao et al., 2008), suggesting that the dorsal ACC is important in processing the sensitivity to the risk of loss. Our findings thus extend prior research by demonstrating that individual differences in stable resting‐state activation of brain areas involved in processing negative outcomes and sensitivity to the risk of loss (ACC) might represent an important source of heterogeneity in risk‐taking behaviour. Consistent with our hypotheses, we found that lower levels of strategic consistency (i.e., higher COV) were associated with decreased resting‐state activation in the left DLPFC. We did not find any associations with insular or VMPFC activation. This finding is in line with previous research suggesting that the primary role of the DLPFC in risky decision‐making might not be a direct involvement in risk‐choices, but rather an indirect involvement through the incorporation of strategic considerations into the decision process (Dockery et al., 2009; Huettel et al., 2005; MacDonald et al., 2000; Steinbeis et al., 2012). Thus, our findings complement prior research by demonstrating that individual differences in stable resting‐state activation of brain areas involved in processing cognitive control and decision‐making (left DLPFC) might represent an important source of heterogeneity in strategic consistency.

Taken together, our study supports a functional dissociation between the ACC and the DLPFC in risky decision‐making (also see MacDonald et al., 2000). Specifically, results suggest that the ACC might be primarily involved in direct, momentary risk‐taking behaviour (also see Clark et al., 2008; Fukunaga et al., 2012; MacDonald et al., 2000; Brown & Braver, 2007, 2008), and the DLPFC might be primarily involved in the long‐term implementation of strategy, providing the basis for risky decision‐making (also see Huettel et al., 2005; MacDonald et al., 2000; Steinbeis et al., 2012). Interestingly, correlation analyses indicated that both RT and COV were associated with both decreased activation of the ACC and DLPFC (see Table S2). Although these results imply that to a certain degree the left dorsal ACC might also be involved in the implementation of strategy, and the left DLPFC might also be involved in actual risk‐taking behaviour, these findings are not further considered as the underlying statistical analyses did not control for multiple testing. Subsequent commonality analyses confirmed this conclusion by demonstrating that the left dorsal ACC accounts for the majority of variance in risk‐taking behaviour, whereas the left DLPFC accounts for the majority of variance in strategic consistency.

Note that our results were based on observations of the delta frequency band and are interpreted according to the assumption that delta oscillations in the resting brain indicate cortical inactivity in specific regions. This assumption is based on different lines of research: First, delta waves have been proposed to have a general inhibitory function, enabling exclusive activation of specific brain regions by inhibiting other brain regions that are not needed for specific tasks (for a review, see Harmony, 2013). Second, delta waves are well known to indicate inactive mental states such as slow‐wave sleep (for a review, see Amzica & Steriade, 1998) or different states of unconsciousness, including anaesthesia (Akeju et al., 2016), coma (Husain, 2006), and propofol‐induced sedation (Lee et al., 2017). Third, delta oscillations have been shown to reflect neural inactivity in previous studies. For example, an anxiety manipulation caused decreased delta activation in the vmPFC, orbitofrontal cortex, and subgenual ACC (Nash et al., 2021), brain regions that have been associated with increased anxiety in previous studies (Greenberg et al., 2013; Straube et al., 2007, 2009; for reviews, see Milad & Rauch, 2007; Shin & Liberzon, 2010). In another study, subjects suffering from melancholia showed increased delta oscillations in the subgenual PFC (Pizzagalli et al., 2004), a brain region that is less active and shows decreased volume in depression (Botteron et al., 2002; Drevets et al., 1997). Based on this framework, we speculate that a greater inhibition of the left dorsal ACC and the left DLPFC at rest as indicated by increased delta oscillations might predispose for increased risk‐taking behaviour and increased strategic consistency, respectively.

In prior research, personality traits have been inconsistently associated with risk‐taking (Becker et al., 2012). Indeed, self‐report is often weakly related to behaviour in general (Dang et al., 2020). Here, in applying a personality neuroscience framework (DeYoung & Gray, 2009), we found that basic personality traits were associated with neural traits linked to heterogeneity in risk‐taking and strategic consistency. Specifically, we found that although only the trait Extraversion was directly related to risk‐taking and strategic consistency, Extraversion, Conscientiousness, Openness to Experience, and Trait Self‐Control were related to decreased resting‐state activation in the left dorsal ACC, and Neuroticism was associated with increased resting‐state activation in the left dorsal ACC. This left dorsal ACC activation in turn mediated an indirect effect of these personality traits on risk‐taking.

The results involving Extraversion and Openness to Experience are consistent with the joint subsystem hypothesis in which neurobiological systems associated with reward and approach motivation are inversely related to neurobiological systems associated with punishment and avoidance motivation, and vice versa (Corr, 2002). Extraversion and Openness to Experience have been characterized as traits related to social and intellectual reward or approach motivation, respectively (DeYoung, 2015; DeYoung & Gray, 2009). Heightened sensitivity to reward, in both the social and intellectual domains, appears to predict muted resting‐state dorsal ACC activation, which then predicts increased risk‐taking. On the other hand, Neuroticism was related to increased resting‐state dorsal ACC activation, which subsequently reduced risk‐taking. This finding is consistent with research demonstrating that Neuroticism is associated with increased sensitivity to negative outcomes (Derryberry & Reed, 1994) and increased ACC activation (Hirsh & Inzlicht, 2008). Conscientiousness and Trait Self‐Control are constructs that are closely related, both theoretically and empirically (*r* = 0.568, *p* < 0.001 in our sample), representing the ability to keep good self‐discipline, resist temptation, and work in a structured manner to achieve long‐term goals (McCrae & Costa, 2008; Tangney et al., 2004). Again, higher abilities in these areas appear to predict muted resting‐state ACC activation, which in turn predicts increased risk‐taking. This is an important finding, as the ACC typically shows *increased* activation during tasks designed to measure self‐control performance (Carter et al., 1998; Holroyd et al., 2004; MacDonald et al., 2000; Van Veen et al., 2001). Combined with our results, higher levels of self‐control and Conscientiousness could be associated with more efficient, task‐dependent functioning of the ACC, which is active in response to appropriate environmental demands (e.g., a self‐control task) but less active at rest (e.g., during a closed‐eyes EEG measurement). Furthermore, the assumption that self‐controlled and conscientious people show a reduced sensitivity to negative outcomes at rest is in line with research demonstrating that both personality traits are positively related to well‐being and negatively related to negative emotion (Anglim & Grant, 2016; Costa & McCrae, 2008; Hayes & Joseph, 2003; Tangney et al., 2004).

Extraversion and Openness to Experience were also associated with decreased resting‐state activation in the left DLPFC, which in turn mediated an indirect effect of these personality traits on strategic consistency. These findings suggest that increased social and intellectual approach motivation predicts muted resting‐state left DLPFC activation, which then predicts more impulsive decision‐making. This finding is in line with associations of Extraversion and Openness to Experience with impulsive behaviour and sensation‐seeking (Aluja et al., 2003; Newman, 1987; Shahjehan et al., 2012; Zuckerman & Glicksohn, 2016) and with studies showing that down‐regulation of the DLPFC leads to more impulsive (and less strategic) decision‐making (Beeli et al., 2008; Cho et al., 2010; Knoch et al., 2006). Together, our results show that different personality traits are related to resting‐state activation of the left dorsal ACC and the left DLPFC and, consequently, heterogeneity in risk‐taking and strategic consistency. More broadly, we propose that personality measures may be more consistently associated with heterogeneity in risk‐taking behaviour if neural traits are taken into account. Note that we did not apply corrections for multiple testing in our correlation and mediation analyses due to the exploratory nature of these investigations. Therefore, future studies including more specific hypotheses are needed to further evaluate our initial personality neuroscience framework of risk‐taking.

In conclusion, the current research examined task‐independent, brain‐based differences to help uncover sources of heterogeneity in risk‐taking and strategic consistency and shed light on why people differ (for reviews, see Braver et al., 2010; Kanai & Rees, 2011; van den Heuvel & Hulshoff Pol, 2010). We find that heterogeneity in risk‐taking is primarily associated with individual differences in resting‐state activation in the left dorsal ACC, and heterogeneity in strategic consistency is primarily associated with individual differences in resting‐state activation in the left DLPFC. Taken together, these results support a functional dissociation of the ACC and DLPFC in the context of risky decision‐making. Furthermore, our results support a personality neuroscience framework (DeYoung & Gray, 2009) by demonstrating indirect links between personality traits and observable behaviour that are mediated by neural traits. Future research could aim to further investigate this framework using other constructs that show weak associations between self‐report and behaviour, such as self‐control, emotional intelligence, and empathy (Dang et al., 2020). We also note that although neural traits are considered to be relatively stable, they are somewhat plastic, or amenable to treatment. For example, paradigms such as neurofeedback, meditation training, or skill repetition can modulate baseline cortical activation or cortical volume in targeted brain regions (Ghaziri et al., 2013; Lazar et al., 2005; Takeuchi et al., 2010). This suggests that different training or neurofeedback paradigms could shape individual differences in left dorsal ACC and/or left DLPFC activation, which may shape individual differences in risk‐taking, or decision‐making processes, more broadly.

## FUNDING INFORMATION

This research received no specific grant from any funding source.

## CONFLICT OF INTEREST

The authors declare that there is no conflict of interest.

## AUTHOR CONTRIBUTIONS

**JL**: Conceptualization, Methodology, Software, Investigation, Writing – Review and Editing. **TK:** Formal Analysis, Data Curation, Writing – Original draft, Visualization. *AT*: Conceptualization, Writing – Review and Editing. **KN**: Conceptualization, Resources, Project Administration, Writing – Review and Editing, Supervision.

### PEER REVIEW

The peer review history for this article is available at https://publons.com/publon/10.1111/ejn.15476.

## Supporting information


**Table S1.** Descriptive statistics of all variables in the study
**Table S2.** Correlations of behavioral measures and personality traits with neural traits
**Table S3.** Regression models controlling for the influence of gender on main results
**Table S4.** Regression models controlling for the influence of experimental conditions on main results

## Data Availability

All code and materials of this study are available upon request (Database: Mendeley; Kleinert, 2021; https://doi.org/10.17632/96bwxvb83m.1).
