# Supplementary material for: Neural signatures of heterogeneity in risk‐taking and strategic consistency
Source: Eur J Neurosci. 2021 Oct 12;54(9):7214–30. doi: 10.1111/ejn.15476 (PMC9292925; doi:10.1111/ejn.15476)
Supplement: Supplementary file 1 — Table S1. Descriptive statistics of all variables in the study Table S2. Correlations of behavioral measures and personality traits with neural traits Table S3. Regression models controlling for the influence of gender on main results Table S4. Regression models controlling for the influence of experimental conditions on main results [file EJN-54-7214-s001.pdf]

Supplementary Material

Neural Signatures of Heterogeneity in Risk-Taking and Strategic Consistency

Josh Leota[1]*, Tobias Kleinert[1]*, Alex Tran[2], Kyle Nash[1]

Running title: Neural Signatures of Risk-Taking

1 Department of Psychology, University of Alberta, Edmonton, Alberta, Canada

2 Institute for Mental Health Policy Research, Centre for Addiction and Mental Health (CAMH), Toronto, Ontario, Canada

* These authors contributed equally to this work

Corresponding author:

Prof. Kyle Nash, knash@ualberta.ca

Phone: +1 780 4928139

Department of Psychology, University of Alberta

P-249 Bio Science – Psychology Wing

11355 – Saskatchewan Drive, Edmonton AB, T6G 2E9

Table S1. Descriptive statistics of all variables in the study

	Min	Max	Mean	SD
**Behavioral measures**				
Risk-Taking (RT)	.13	12.68	3.91	2.72
Coefficient of Variability (COV)	.16	.89	.47	.13
**Personality traits**				
Extraversion	1	5	3.00	1.06
Agreeableness	1	5	3.60	.70
Conscientiousness	1	5	3.82	.81
Neuroticism	1	5	2.92	.96
Openness to Experience	1.5	5	3.66	.76
Trait Self-Control	1.38	4.54	3.01	.66
**Neural traits**				
Delta density left dorsal ACC (μV^2)	-2.41	1.54	.27	.76
Delta density left DLPFC (μV^2)	-1.24	3.73	1.15	.83

N = 104. Min = minimum value, Max = maximum value, Mean = mean value, SD = standard deviation. Risk-Taking (RT) and the Coefficient of Variability (COV; inverse index of strategic consistency) were measured with the Balloon Analogue Risk Task (BART; Lejuez et al., 2002), the Big 5 personality traits were measured with the Ten Item Personality Inventory (TIPI; Gosling et al., 2003) and Trait Self-Control was measured with the Self-Control Scale (Tangney et al., 2004).

Table S2. Correlations of behavioral measures and personality traits with neural traits

	**Delta density left dorsal ACC**		**Delta density left DLPFC**	
	**r**	**p**	**r**	**p**
**Behavioral measures**				
Risk-Taking (RT)	**.317**	**.001**	**.241**	**.014**
Coefficient of Variability (COV)	**.238**	**.015**	**.326**	**.001**
**Personality traits**				
Extraversion	**.313**	**.001**	**.222**	**.024**
Agreeableness	-.109	.270	-.060	.544
Conscientiousness	**.229**	**.020**	.172	.082
Neuroticism	**-.276**	**.005**	-.171	.083
Openness to Experience	**.281**	**.004**	**.228**	**.020**
Trait Self-Control	**.224**	**.022**	.097	.329

N = 104. r = Pearson correlation coefficient, p = p-value. Bold values indicate significant correlations (two-sided, alpha level = .05). Pearson correlations for associations of the behavioral measures RT (Risk-Taking) and COV (Coefficient of Variability) obtained from the Balloon Analogue Risk Task (BART; Lejuez et al., 2002) and personality traits (Big 5, obtained from the TIPI; Gosling et al., 2003; and Trait Self-Control, obtained from the SCS; Tangney et al., 2004).

Table S3. Regression models controlling for the influence of gender on main results

**Model 1: RT + Gender → Delta density left dorsal ACC**				
	β	**t**	**p**	**Model R^2**
Risk-Taking (RT)	.315	3.40	.001	
Gender	-.182	-.197	.052	.116
**Model 2: COV + Gender → Delta density left DLPFC**				
	β	**t**	**p**	**Model R^2**
Coefficient of Variability (COV)	.289	3.13	.002	
Gender	.228	2.46	.016	.140

N = 104. *β* = standardized regression coefficient, t = t-value of predictor, p = p-value of predictor, Model R^2 = adjusted R^2 of the overall model. Regression models showing the influence of gender on the main results of this study. When adding gender as a predictor to a model regressing delta density in the left dorsal ACC on RT (Model 1), both RT (p = .001) and gender (p = .052) contribute to an increased model fit (*adjusted* R^2*change* = .025). Subsequent correlation analyses reveal that women show a slightly stronger association of RT and delta density in the left dorsal ACC ($r(58)$ = .357, p = .005) compared to men ($r(42)$ = .271, p = .075). However, these correlations don't differ significantly from each other (*Fisher's z* = -.466, p = .320). Similarly, when adding gender as a predictor to a model regressing delta density in the left DLPFC on COV, both COV (p = .002) and gender (p = .016) contribute to an increased model fit (*adjusted* R^2*change* = .043). Subsequent correlation analyses reveal that men show a slightly stronger association of COV and delta density in the left DLPFC ($r(42)$ = .345, p = .022) compared to women ($r(58)$ = .287, p = .026). Again, these correlations don't differ significantly from each other (*Fisher's z* = .315, p = .376).

Table S4. Regression models controlling for the influence of experimental conditions on main results

**Model 1: RT + Condition → Delta density left dorsal ACC**				
	β	**t**	**p**	**Model R^2**
Risk-Taking (RT)	.313	3.31	.001	
Condition	-.057	-.608	.545	.086
**Model 2: SB + Condition → Delta density left DLPFC**				
	β	**t**	**p**	**Model R^2**
Coefficient of Variability (COV)	-.314	-3.24	.002	
Condition	-.049	-.508	.613	.091

N = 104. *β* = standardized regression coefficient, t = t-value of predictor, p = p-value of predictor, Model R^2 = adjusted R^2 of the overall model. Regression models showing that the main results of this study remain significant, and at similar levels, when controlling for the influence of experimental conditions (anxiety or control; manipulation unrelated to the purpose of this study). When adding condition as a predictor to a model regressing delta density in the left dorsal ACC on RT (Model 1), RT remains the only significant predictor ($p = .001$). The overall model fit decreases, without changing significantly (*adjusted* R^2*change* = -.006). Similarly, when adding condition as a predictor to a model regressing delta density in the left DLPFC on COV (Model 2), COV remains the only significant predictor with ($p = .002$). Again, the overall model fit decreases, without changing significantly (*adjusted* R^2*change* = -.007).
